# Supplementary material for: Are carbon emissions trading and green financial instruments synergistic? -Comprehensive quantitative research based on content analysis
Source: PLoS One. 2024 Mar 7;19(3):e0298601. doi: 10.1371/journal.pone.0298601 (PMC10919601; doi:10.1371/journal.pone.0298601)
Supplement: S1 Data — (ZIP) [file pone.0298601.s001.zip › Data/地级市Z_F工作报告.zip!/╔╜╬≈/╨├╓▌╩╨/└┤╫╘╣1⁄2╓┌║┼í╛┬φ┐╦╩2╛▌═°í┐_2016.docx]

各位代表：

　　现在，我代表市人民政府，向大会报告政府过去五年的工作，对《忻州市国民经济和社会发展第十三个五年规划纲要（草案）》作简要说明，报告今年工作和建议，请予审议，并请市政协委员和列席人员提出意见。

　　一、过去五年工作

　　过去的五年，是忻州经济社会发展进程中极不平凡的五年。五年来，面对艰巨繁重的改革发展稳定任务，面对经济下行压力持续加大的复杂严峻局面，面对经济新常态下的种种风险挑战，面对人民群众寄予的殷切期盼，我们始终坚持以习近平总书记系列重要讲话精神为指引，认真贯彻落实省委、省政府决策部署，在市委坚强领导下，坚持“产业第一、项目至上、企业为重、服务为本”，顶住各种困难和压力，抢抓一切有利机遇，着力推动全市经济社会发展在攻坚克难中浴火重生、砥砺前行。各项预期性指标总体完成，各项约束性指标全面完成，“十二五”规划的目标任务基本实现。

　　这五年，是我们坚持发挥后发优势，努力奋勇赶超，综合实力跨上新台阶，主要经济指标进位争先的五年。

　　不断引深项目建设攻坚战，充分发挥投资在稳增长中的关键作用。坚持“八位一体”重点工程推进机制，对重大产业项目实行分类考核，引导各级干部在项目建设主战场上比实绩、论英雄。全社会固定资产投资累计完成4069.5亿元，是“十一五”时期的3.7倍；年均增长21.4%，高出全国、全省6.3个、4.1个百分点；2015年突破千亿大关，达到1120亿元，是2010年的2.6倍。

　　千方百计扩大居民消费，切实发挥消费在稳增长中的基础作用。积极鼓励汽车、家电、装修等大宗消费，有力促进养老家政、文化娱乐、旅游观光等现代消费，大力倡导节能环保等绿色消费，着力发展电子商务等新型业态，全市社会消费品零售总额比“十一五”时期翻了一番。

　　着力服务企业生产经营，积极发挥企业在稳增长中的主体作用。认真落实省政府煤炭、煤层气、低热值煤发电3个“20条”和减轻企业负担“60条”，出台系列支持企业发展政策措施，在“减、免、缓、帮、扶、替”上下功夫，为企业减负31亿元，其中2015年达27亿元，稳住了经济增长的基本面。

　　2015年，全市GDP完成681.2亿元，比2010年增加239.7亿元，年均增长9%，在全省占比由4.8%提高到5.3%；一般公共预算收入完成73.7亿元，比2010年增加31.4亿元，年均增长11.7%，在全省占比由4.4%提高到4.5%；规模以上工业 增加值完成255.1亿元，比2010年增加89.8亿元，年均增长12.5%，在全省占比由3.7%提高到6%；主要经济指标彻底摆脱了全省垫底局面，增速位次连年靠前，总量稳定前移，占比不断提高。全市经济社会发展进入了赶队前行的历史新时期。

　　这五年，是我们坚持综改统领，推进潜能转化，经济结构不断优化，板块发展初步成型的五年。

　　新型综合能源基地已现雏形。2015年原煤产量6117万吨，是2010年的近2倍。着力推进煤电一体化，大力开发新能源。全市电力总装机913.1万千瓦，较“十一五”末增加391.7万千瓦。其中，火电建成投产496万千瓦，核准在建和取得“路条”项目606万千瓦，共计1102万千瓦，装机总量全省第二，是“十一五”末的3.6倍。新能源发电装机417.1万千瓦，总量全省第一，较“十一五”末实现翻番；新增风电装机182万千瓦，达到197万千瓦，占全省风电总装机的近1/3；光伏发电装机达到11万千瓦，占到全省的12%。垃圾发电、生物质能发电、燃气产业发展填补空白。

　　非煤产业取得实质性突破。2015年氧化铝产量达287万吨，是2010年的2.2倍；金宇超细煅烧高岭土、久力新型建材等一批新材料形成规模。法兰产业加快兼并重组、技术升级，产品延伸到塔筒等部件和整机，整合后的锻造企业2015年产量达34.8万吨，占全县的一半以上，定襄县被授予国家外贸转型升级专业型示范基地；德奥电梯建成投产；阳煤忻通、美新通用等煤机装备制造业集群发展。煤炭运销等物流产业加速发展，成为神池、五寨、岢岚、偏关等县财政收入的重要渠道。现代农业加快发展，粮食连续4年获得丰收，总产稳定在30亿斤以上，忻州市被授予“中国杂粮之都”、岢岚县被授予“中华红芸豆之乡”、静乐县被授予“中国藜麦之乡”、神池县被授予“中国亚麻油籽之乡”、五寨县被授予“中国甜糯玉米之乡”。旅游产业快速发展，五台山景区管理体制改革完成，雁门关、芦芽山申报5A景区通过初验，忻州云中河景区成功创建国家4A级旅游景区，老牛湾、河边村等入选“山西最美旅游乡村”，2015年全市旅游总收入达284.6亿元，年均增长21.8%。文化体制改革活力显现，忻州被评为“全国文化体制改革工作先进市”。全市非煤产业投资占工业投资比重由2010年的72.5%提高到2015年的79.7%，非煤产业增加值占工业增加值比重由62.1%提高到66.4%。

　　产业集聚发展步伐加快。以循环园区、特色园区为载体，集聚关联企业，进一步放大成本优势、规模优势。全市共建设产业集聚区15个，落地开工项目189个，基础设施建设投入25亿元。忻州开发区、定襄、五台、岢岚“飞地经济”率先起步，原平经济技术开发区列入省级开发区。

　　建设三个门户、打造三个集散地、发展六大经济板块的“336”综改布局全面推进。保德县、河曲县在“保府经济圈”中崭露头角，忻府区、定襄县、原平市多元经济板块日趋壮大，代县、繁峙县通过灵河高速实现与北京、天津的互联互通。东部五台山，中部奇村、顿村温泉度假区，西部芦芽山“三个集散地”功能逐步显现。县域要素整合、联动发展进入快车道。2014年全省县域经济发展考核评价，我市C类县排名总体靠前，河曲县获C类县“状元”。

　　这五年，是我们坚持基础先行，做实投资硬件，城乡面貌焕然一新，对外形象发生深刻变化的五年。

　　加快市域城镇化进程。中心城市带头，县级城镇同步，持续大干城建、提升城市管理水平。忻州城区新建改造道路88条，全长142.86公里，形成“九横十一纵”的城市路网，城市规划、建设、管理走上法治轨道。12个县（市）成功创建国家卫生城市；城市公共设施明显改善，建成区绿化覆盖率、集中供热普及率均提高15个百分点；建成城镇保障性住房9.67万套，全市常住人口城镇化率年均提高1.69个百分点。忻州市被确定为“国家可再生能源建筑应用示范城市”、“智慧城市试点市”。

　　着力改善农村人居环境。完成新一轮农村“五个全覆盖”工程，办好农村“五件实事”，努力巩固两轮“五个全覆盖”工程成果。实施农村人居环境改善工程，解决了2014个村、76万农村人口和6.4万农村学校师生的饮水安全问题，易地搬迁特困群众13.2万人，改造农村困难家庭危房41863户，农村人均住房面积27.4平方米。

　　统筹推进重大基础设施项目。大西高铁加快推进，五保、环城、神河、繁大高速建成运营，五盂、原神高速建成，神岢高速、晋蒙黄河大桥开工，高速公路新增通车里程355公里，全市公路密度由66.2公里/百平方公里提高到68.59公里/百平方公里；五台山机场建成通航。新增农田实灌面积56.25万亩，实现农民人均近1亩水浇地。实施新一轮农网改造，农电配电网投资是“十五”和“十一五”期间总和的2倍；蒙西—天津南交流、晋北—江苏直流工程有序推进，境内第二座500千伏变电站（五寨）建成，在运220千伏变电站达到14座，为新型综合能源基地建设提供了坚强保障。

　　加快建设美丽忻州。淘汰落后钢铁产能20万吨、水泥产能276万吨、铁合金产能9.97万吨、焦化产能40万吨，万元地区生产总值综合能耗下降18.12%，万元工业增加值用水量下降59.6%；电力、水泥等重点行业脱硫、脱硝、除尘改造任务全部完成，淘汰黄标车、老旧车32117辆，全市主要污染物排放总量明显下降，繁峙、神池成功创建省级环境保护模范城市。五年营造林320.17万亩，森林覆盖率提高4.1个百分点，2015年全省造林绿化现场会在我市召开；汾河流域生态修复治理全面启动。

　　这五年，是我们坚持全面深化改革，扩大对外开放，推动大众创业、万众创新，发展动力和活力显著增强的五年。

　　重点领域、关键环节改革取得实效。煤炭管理体制改革迈出重大步伐，涉煤收费项目全面清理规范，煤炭资源税实行从价计征，煤焦公路销售所有行政授权、运销票据、检查站点全部取消。中国（太原）煤炭交易中心忻州交易处启动运营，成为全省首家在地市设立的交易机构。率先在全省推进收费制度改革，实现了收缴分离、票款分离、权钱分离。坚持一企一策，运用“人资分离”等办法，成功推进云河集团、钨丝厂等市属国有企业改制；支持民营企业建设管理团队、营销团队、技术团队，向现代企业跨越。扎实推进商事制度改革，年均新登记企业数量是改革前的2倍。集体林权主体改革任务基本完成，农村土地承包经营权确权登记颁证全面展开，土地承包经营权抵押贷款试点顺利推开，山西省产权交易中心入驻忻州。

　　“三个突破”强力推进。加快金融振兴，“助保贷”实现全覆盖，设立企业资金链应急周转保障资金，推进企业完善资产凭证，搞好抵押物储备，不断做实信用基础；交通银行、晋商银行落户忻州，7家村镇银行在县域设立，忻州农商行、五台农商行挂牌成立；永安信（忻州）股权投资资金成功揭牌。2015年全市金融机构余额存贷比比2010年提高7个百分点。加快科技创新，国家级企业技术中心实现零突破，建成省级企业技术中心15户、市级企业技术中心68户、省级重点实验室1户，涌现出高新技术企业25家；组织开展了科学技术奖评比活动，企业“五小”活动掀起新高潮。加快民营经济发展，出台扶持民营经济发展的“35条”和支持小型微型企业发展的“14条”，从2015年起每年安排3000万元全民创业专项扶持资金和3000万元中小企业发展专项资金；加快资源型企业转型项目建设，新增年营业收入超亿元的“小巨人”企业46个。举办了忻州市首届青年创新创业大赛，e谷忻州市大学生创业园区建成投运。第五届中国创新创业大赛（山西赛区）颁奖典礼暨项目展示活动在我市成功举办。

　　对外开放水平进一步提升。积极融入太原都市圈、京津冀和环渤海经济圈，主动承接长三角、珠三角地区产业转移，成功加入环渤海区域合作市长联席会；抓住中博会、央企山西行等重大商洽活动契机，突出精准招商、产业链招商，开展“迎老乡、回故乡、建家乡”活动，招商引资签约项目到位资金2046亿元，是“十一五”时期的5.1倍，忻州市跻身2015年中国宜商城市竞争力前200名城市的第158名。

　　这五年，是我们坚持执政为民，努力造福群众，切实保障和不断改善民生的五年。

　　大力发展社会事业。岢岚县、保德县通过国家义务教育均衡发展评估认定，新建改扩建标准化公办幼儿园606所，新建忻州高级中学、长征小学、实验幼儿园，县级公立医院综合改革全面推开，政府办基层医疗卫生机构和村卫生室全部实行基本药物制度，忻州市人民医院建成投用，市中医院改扩建完成，市儿童医院主体完工。市级“五馆一院”加快建设，全市公益性文化设施全部免费开放，政府购买公共演出服务全面推行，《情悟五台山》等25部优秀作品获得国家和省级奖项。忻州市连续七届荣膺“全国双拥模范城”。

　　切实关心群众生活。城镇累计新增就业18.4万人，转移农村劳动力37.85万人，城镇登记失业率控制在4.2%以内。连续提高最低工资标准，机关事业单位工作人员工资标准与省直机关拉平，城乡居民人均可支配收入年均增长11.4%、13.7%。大力推进扶贫开发，五年累计脱贫41.7万人。城乡居民基本养老保险制度统一实施，连续提高企业退休人员基本养老金水平，机关事业单位养老保险制度改革稳步推进，城乡居民医保财政补助标准、城乡低保标准、农村五保对象补助标准按政策兑现，城乡社会救助实现全覆盖。

　　维护社会安全稳定。认真落实“4438”工作机制，促进安全生产形势向稳定好转的目标迈进。忻州市连续四年被评为“全省安全生产模范市”；煤矿百万吨死亡率控制在0.08%以下，远低于全国、全省同期水平，2015年实现“零死亡”。加强和创新社会治理，扎实推进“平安忻州”建设，9个县被评为省级平安县；注重社会热点问题引导，及时解决群众切身利益问题。全面完成食品药品监管体制改革任务，基层监管能力进一步增强，保障了群众“舌尖上的安全”。

　　这五年，是我们坚持转变职能，建设“六型政府”，着力践行和弘扬忻州政府精神，形成干事创业良好导向的五年。

　　始终坚守“执政为民、敢为人先、群策群力、滴水石穿”的忻州政府精神，认真落实“牢记宗旨、甘当公仆，勤奋学习、刻苦工作，恪尽职守、敢讲真话，依法办事、从严治政，严于律己、清正廉洁”的“五章约法”，制定实施政府建设三年规划和年度行动计划，努力建设法治、为民、服务、效能、诚信、廉洁政府。

　　严格依法办事。自觉接受人大及其常委会的监督，积极支持人民政协履行职能，办理人大代表建议617件、政协提案1465件。开展“给人民汇报、请人民评议、向人民问计”政风行风活动，“忻州随手拍”被评为全国政务新媒体优秀公众号、第八届中国政府创新最佳实践奖和全国优秀决策案例奖。完成第九届、第十届村委会换届选举和“六五”普法任务。

　　加快职能转变。市级政府部门权力清单、责任清单全部按期公布，市级政务服务平台、公共资源交易平台和政务服务网络加快建设，新一轮政府机构改革全面完成。坚持简政放权、放管结合、优化服务，取消、下放和调整行政审批事项296项。

　　持续改进作风。认真执行中央“八项规定”精神，自觉践行“三严三实”，严格落实国务院“约法三章”，坚持不懈纠正“四风”，深入开展党的群众路线教育实践活动和“学习讨论落实”活动，市级党政机关公务用车制度改革基本完成，“三公”经费大幅压减，节省的经费全部用于民生。

　　坚持从严治政。教育引导政府工作人员“理政、谋政、勤政、善政、廉政”，严格落实“两个责任”，始终保持反腐败高压态势，一批群众反映强烈的突出问题得到解决，凝聚了人心，锻炼了队伍，造就了一种不甘人后、奋起直追的好风气，进一步赢得了群众对政府工作的支持。

　　各位代表，过去五年的成绩来之不易。这是省委、省政府坚强领导、亲切关怀的结果，是市委统揽全局、科学决策的结果，是市人大、市政协大力支持、有效监督的结果，是全市广大干部群众同心同德、团结奋斗的结果。在此，我代表市人民政府，向全市人民，向各位人大代表、政协委员，向驻忻解放军、武警官兵、公安民警和驻忻单位，向所有关心、支持、参与忻州发展进步的朋友们，致以崇高的敬意和衷心的感谢！

　　我们清醒认识到，我市经济社会发展还存在不少困难和问题。工业企稳回升基础脆弱，各级财政运行趋紧。经济总量小、民生欠账多、城镇化水平低的状况仍未根本改变；传统产业产能过剩，新型产业发展不足，资源型经济偏重的问题仍然突出；贫困面大，脱贫攻坚任务艰巨，全面建成小康社会亟需付出艰苦努力；安全生产形势依然严峻，经济下行导致社会稳定潜在风险和隐患增多；政府建设和干部作风与人民群众期盼还有一定差距。

　　我们深切体会到，发展是第一要务，所有问题要用发展的办法来解决，必须以经济建设为中心，咬定青山不放松，一门心思抓发展。改革是第一动力，必须坚持问题导向，向改革创新要答案，使改革创新成为解决问题、推动发展的强劲引擎。民生是第一关切，必须始终高举依法办事和依靠群众两面旗帜，运用法治思维和法治手段推动工作，真正把群众作为衣食父母，办好事关群众切身利益的每一件事情。干部是我们事业的骨干，必须把对干部的严格要求作为对干部最好的关心爱护，靠带动、示范、坚守，推动政府工作人员作风转变，形成“九牛爬坡、个个出力”的干事创业局面。实现忻州振兴崛起，关键在于我们自己，必须立足实际，一切从自己身上找原因，一切立足自己解决问题，靠苦功夫、笨办法，孜孜以求，久久为功，始终保持力争上游的态势，为忻州长远发展夯基础，最终实现由量变到质变的飞跃。

　　总之，我们要通过艰苦细致的工作和坚持不懈的努力，认真解决发展中遇到的困难和问题，努力谱写忻州发展史上的新篇章！

　　二、“十三五”时期的目标任务

　　《“十三五”规划纲要（草案）》已印发各位代表，这里作一简要报告。

　　《纲要草案》紧紧围绕全面建成小康社会奋斗目标，强调要牢固树立和贯彻落实新发展理念，实施“1661”发展战略，明确了今后五年经济社会发展的主要目标任务，突出了以下9个方面：

　　经济保持中高速增长，产业发展迈向中高端水平。到2020年实现地区生产总值和城乡居民人均收入比2010年翻一番，“十三五”时期经济年均增长保持在7%左右。努力推动产业转型升级取得明显进展，投资效率和企业效益明显上升，消费对经济增长的贡献率明显加大，新型综合能源基地建设取得新突破。

　　坚持脱贫攻坚统揽，打赢脱贫攻坚战。贯彻精准扶贫、精准脱贫基本方略，创新扶贫工作机制和模式，全面实施“3659”脱贫攻坚策略，到2020年全市11个贫困县如期摘帽、2222个贫困村整体出列、35.33万贫困人口全部脱贫。

　　实施创新引领发展，着力提高发展质量和效益。继续坚持“产业第一、项目至上、企业为重、服务为本”，持续推动大众创业、万众创新。加强供给侧结构性改革，努力做好煤与非煤两篇文章，加快工业强基、文旅兴市和“三农”现代化步伐。促进大数据、云计算、物联网广泛应用。研究与试验发展(R&D)经费年均增长23.6%以上，科技进步对经济增长的贡献率显著增强。

　　实施协调持续发展，着力形成平衡发展结构。更加注重围绕改善民生来谋划发展，推动经济社会协调发展。加快忻定原城镇组群发展，促进区域协调发展。实施新型城镇化发展战略，全力推动“五城联创”，推动城乡协调发展，到2020年常住人口城镇化率达54.81%。

　　推进绿色永续发展，着力建设美丽忻州。坚持在发展中保护、在保护中发展。深入实施大气、水、土壤污染防治行动计划，加强生态保护和修复，构筑生态安全屏障。推动低碳循环发展，促进资源节约和高效利用。单位国内生产总值用水量、能耗、二氧化碳排放量持续下降，森林覆盖率达到21%，生态环境质量总体改善。

　　实施开放融合发展，着力实现合作共赢。紧抓国家“一带一路”、京津冀区域一体化等重大战略机遇，深化开放活市战略，建设“三个门户”，发展外向型产业园区，加快对外贸易优化升级，提升招商引资水平，构建开放发展新格局。

　　实施共享发展，着力保障和改善民生。坚持以人民为中心，持续增进民生福祉。完善社会保障体系，实施义务教育学校标准化、高中阶段教育普及工程。坚持就业优先，推进健康忻州建设，促进人口均衡发展。加大棚户区改造力度，完善住房保障体系，到2020年城镇人均住房建筑面积达35平方米以上。

　　实施安全发展，着力营造良好社会氛围。坚持全面从严治党，持续推进党风廉政建设和反腐败斗争，构建良好政治生态。严格落实安全生产责任制，推进社会治理精细化，构建共建共享社会治理新格局。预防和化解社会矛盾，大力推进平安忻州、法治忻州建设。

　　全面深化改革，为全面小康建设提供持续动力。加快转型综改试验区建设，深化现代产权制度、国有企业、要素市场、价格机制、财税体制、行政管理体制等重点领域和关键环节改革，建立、完善实现全面脱贫全面小康目标的政策体系和体制机制。

　　各位代表，《纲要（草案）》经本次会议审议通过后，市政府将全力抓好落实。我们坚信，通过全市人民的共同努力，我市国民经济和社会发展第十三个五年规划确定的奋斗目标一定能够实现！

　　三、2016年工作

　　2016年，是实施“十三五”规划的开局之年，也是推进供给侧结构性改革的攻坚之年。做好政府工作，要高举中国特色社会主义伟大旗帜，深入学习贯彻习近平总书记系列重要讲话精神，遵循“五位一体”总体布局、“四个全面”战略布局和新发展理念，坚持中国特色社会主义政治经济学重大原则，按照省委“一个指引、两手硬”的重大思路和要求，主动适应经济发展新常态，把深化供给侧结构性改革和建设转型综改试验区紧密结合起来，全力实施“1661”发展战略，即以脱贫攻坚统揽经济社会发展全局，以产业转型、三区联动、五城联创、交通先行、民生改善、法治建设为重点，推进创新发展、协调发展、绿色发展、开放发展、共享发展、安全发展，团结带领全市人民,对标前行，苦干实干，为与全省同步建成全面小康社会不懈奋斗。

　　主要预期目标是：地区生产总值增长6.5%以上，全社会固定资产投资增长15%以上，社会消费品零售总额增长6%左右，公共财政预算收入下降0.5%，城镇常住居民人均可支配收入增长7%，农村常住居民人均可支配收入增长7.1%。城镇新增就业3.7万人，登记失业率控制在4.2%以内，居民消费价格涨幅控制在3%。

　　约束性指标是：万元地区生产总值综合能耗降低3.2%，二氧化硫排放量削减4%，化学需氧量排放量削减3.52%，氮氧化物排放量削减4%，空气质量优良天数比例71.4%，劣v水体比例0%，农村贫困人口脱贫8万人，城镇棚户区住房改造12000套。万元地区生产总值二氧化碳排放量、万元地区生产总值用水量、新增建设用地规模完成省下达任务。

　　重点抓好以下10个方面的工作：

　　（一）强化创新引领和综改试验，增强经济发展内生动力。

　　推进观念创新。树立新型的开放观，运用“地球村”的眼光和方式认识、思考问题。树立新型的市场观，发挥好“无形的手”的作用，以销为先，市场主导。树立新型的竞争观，做到人有我优，彰显特色。树立新型的法治观，运用法治思维和法治手段解决问题、推动工作、提升水平、促进发展。树立新型的就业观，深化自主择业，以创业带动就业。树立新型的人才观，只要为忻州发展作出贡献的，只要在社会上通过合法手段拥有财富的都是人才。

　　推进业态创新。改造提升传统动能，促使潜在能量转化成现实产业，资源优势转化成产业优势，区位优势转化成商贸物流产业，市场需求转化成现实生产力。以企业联盟、企业集团、企业合作、企业兼并为主，创新企业形态。运用信息网络等现代技术，推动生产、管理和营销模式变革，推进管理模式创新、经营模式创新、创业模式创新。发挥好大众创业、万众创新和“+互联网”集众智汇众力的乘数效应。

　　推进科技创新。实施“高新技术企业育种工程”，努力培育一批具有自主创新能力的企业技术中心，新建一批产业技术创新战略联盟。加快发展科技服务业，深入开展“五小”专项活动，实施“一园十站双百聚才计划”，推进产学研用深度融合。深化科技管理体制改革，推动政府职能从研发管理向创新服务转变。实施知识产权发展战略，加大知识产权保护力度，促进有效发明专利拥有量稳步增长。

　　推进金融创新。完善银企对接机制，用好企业资金链应急周转保障资金，帮助企业解决实际困难。引进银行业金融机构，推动神池、五寨、岢岚、静乐、河曲5县设立村镇银行；完成繁峙农商行挂牌，河曲、神池、岢岚农信社改制农商行筹建，启动原平、代县农信社改制农商行，加快定襄农信社改制。对接多层次资本市场，实现我市企业在全国中小企业股份转让系统挂牌“零突破”；用好股权融资、中小企业集合票据融资等直接融资手段，扩大企业直接融资比例。规范小额贷款公司经营，推进互联网金融发展。做实政策性融资担保公司，发挥混合所有制担保公司的应有作用。推动险资入忻，落实“三农”保险，力争保险密度接近全省年均水平。防范和处置各类金融风险，不断优化金融生态环境。

　　（二）推进供给侧结构性改革，加快产业转型升级。

　　推进企业兼并重组。打好国有企业提质增效攻坚战，积极稳妥处置“僵尸企业”，推进市属国有企业改制，加快云瑞商贸、云马焦化破产终结，抓紧轻工业品经销公司债权追索，推动云河集团北区土地联合开发，深化水务公司改革，启动钨丝厂破产，实施五交化重组。做好企业改制和破产后续工作，完成企业社会职能移交。转变国有资产监管职能，实现国有资产保值增值。利用市场倒逼机制推动神达集团和阳煤集团的合作，实现由煤的合作、铝的合作向煤化工的跨越。

　　建立健全现代企业制度。完善法人治理结构，规范市属国有企业董事会制度和运行机制。充分发挥监事会作用，增强监督的及时性、有效性。加快推动民营企业建立现代企业制度，实现由家族式、作坊式企业向现代企业的跨越。

　　精准帮扶企业。全面落实“降成本”政策措施，继续在“减、免、缓、帮、扶、替”上下功夫，努力把企业不必要的制度性交易成本降下来。加强企业内部管理，提升市场竞争能力。推动现有企业转化升级，培育新的经济增长点。

　　激发民营经济活力。鼓励民营企业扩大投资、参与国有企业改革，在项目核准、融资服务、财税政策、土地使用等方面做到一视同仁，依法保护各种所有制经济产权，营造公平、公正、透明、稳定的法治环境。年内建成1个市级中小企业综合服务平台，新增10个以上“小升规”企业、1000个中小微型企业。用好国家对贫困县企业IPO或挂牌“新三板”“即报即审、审过即发”政策，引导我市优势企业进军资本市场，加快推进企业上市（挂牌）。大力发展总部经济，切实打造金融洼地、发展热土，着力吸引符合条件、有战略眼光的企业来忻发展，依托资本市场不断发展壮大地方经济的实力。

　　推动煤炭行业脱困转型。落实“去产能”部署，严控增量，主动减量，优化存量，努力巩固和拓展煤炭市场份额；稳定煤炭物流产业。积极实施煤电一体化战略，投运广宇二期，加快晋能保德、华润宁武、山煤河曲等电厂建设。

　　促进能源产业高效发展。深化电力体制改革。在积极推进大用户直供电基础上，抢抓直接交易的政策红利，降低电力用户的能源成本，提高发电企业的利用小时，提升我市电力工业发展能力。加快大唐山西新能源岢岚40万千瓦等5个风电项目建设，推进一批光伏发电项目。推进保德海通杨家湾50万方煤层气液化项目、山西汽运唐子梁煤层气液化项目，努力使煤层气开采成为我市新能源建设的新亮点。

　　推动现代载能产业绿色发展。发展煤电铝材一体化，加快国电投山西铝工业和同德铝工业循环经济园区项目建设，基本实现氧化铝产能就地转化。加快推进定襄取向硅钢项目建设。积极发展低能耗新型材料。

　　做强做大战略性新兴产业。围绕“中国制造2025”山西行动纲要和全省新兴制造业三年推进计划，做大做强铸造锻造产业，走精加工的路子；扶持阳煤忻通等装备制造重点企业，加快中科忻能等新能源电动汽车产业发展，推动管家营等法兰企业加强技术改造，实现配件向装备的跨越。加快推进天宝等风电装备制造项目，力争培育5—10户年产值超过3亿元的节能环保型装备制造企业。启动实施以机场为核心的空港产业园建设。以五台山风景区为龙头，加快以三个集散地、五大旅游板块为重点的文化旅游产业发展，依法建立健全旅游机构，扎实推进景区景点经营体制改革，认真实施“五个一批”，加快芦芽山、雁门关、阎锡山故居5A景区创建，不断深化乡村旅游，抓紧推出全市旅游发展基金，尽快把文化旅游产业培育成忻州的战略性支柱产业，建设文化旅游强市。加快永旺物流、忻州汽贸文化园等项目建设，大力发展养老、医疗、健康、家政等服务业，提高服务业在GDP中的比重。

　　（三）引深项目攻坚，增强经济发展后劲。

　　加强项目策划和储备。按照“有”转“优”、“无”转“有”、“农”转“工”、“散”转“聚”、“短”转“长”的思路，发挥优势，研究政策，提升项目策划和储备的针对性。注重对产业项目、预算类资金项目、基金类项目、经营性企业项目目录的分类管理，加强对接，加快投资，有力拉动经济增长。

　　发挥有效投资对稳增长调结构的关键作用。抓好9大重点领域，计划总投资415.9亿元。其中：铁路项目11项、投资27.2亿元；公路项目2项、投资21亿元；低热值煤发电项目7项、投资59.7亿元；水利项目22项、投资6亿元；城乡人居环境改善项目45项、投资35.6亿元；煤层气项目2项、投资6亿元；节能环保项目8项、投资6.8亿元；新兴产业项目337项、投资253.6亿元。推进省市重点工程建设，项目储备9247亿元，项目签约1534亿元，项目落地1039亿元，项目开工835亿元，省市县重点工程投资945亿元，项目投产952亿元。拓展市汇丰公司、城投公司平台作用，争取一批基金项目，抓好一批政府购买服务项目。

　　实施重大产业项目分类考核。忻州经济开发区和忻府、原平、河曲、保德、宁武6个A类县（市、区），新开工10亿元以上产业项目2个、5亿元以上产业项目3个、3亿元以上产业项目4个；繁峙、代县、定襄、静乐、五台5个B类县，新开工10亿元以上产业项目1个、5亿元以上产业项目3个、2亿元以上产业项目5个；五寨、岢岚、神池、偏关、五台山景区等5个C类县（区），新开工5亿元以上产业项目2个、3亿元以上产业项目3个、1亿元以上产业项目4个。

　　提升对外开放和招商引资水平。把五台山机场航空口岸开放作为对外开放的重点。把握环渤海经济圈建设、黄河几字湾经济合作等历史机遇，发挥资源、区位、环境优势，深化交流合作。突出补链、循环、提升、扩张、延伸、转化，依托企业和产业，创新招商引资方式，在招引世界500强和国内500强上实现突破。抓好民企入晋项目落地。引深“迎老乡、回故乡、建家乡”活动，推动忻商回乡、民企入忻，努力把忻州打造成投资洼地和创业乐土。

　　总结完善项目建设有效机制。坚持“产业第一、项目至上、企业为重、服务为本”，继续实行“八位一体”，严格落实政府推进、部门服务、企业实施“三个主体责任”，向上争取，向下服务，不断提高土地、环评、立项、规划、许可等各个环节的服务水平，推动项目建设不断取得新成效。

　　切实打好“双百”行动攻坚战。扎实推进项目建设百日攻坚行动和工业经济百日攻坚行动，开展“各类申报项目受理材料大起底”“万名干部入企业服务活动”。坚持依法和创新相结合，有效解决项目建设中的困难和问题，加快项目落地。实施增长点工程，释放国家和省降低实体经济企业成本的政策红利，着力提升企业的竞争力。

　　（四）优化发展格局，加快集聚发展。

　　持续改善城乡人居环境。启动实施“五城联创”，全面推进忻州城区南部改造、中部提升、北部开发，实施垃圾发电、排洪、燃气管网改造等市政基础设施项目，加快广宇煤电二期项目供热配套设施建设；忻州城区30条道路建设和改造工程国庆节前如期竣工通车，市区公交经营体制改革稳步推进，144台纯电动公交车上线运营，忻州城区创建省级园林城市工作加快推进；大力推动“忻定原”同城化，启动忻原大道、忻定大道、机场高速连接线建设，实施农村旅游公路规划项目建设，规划建设108、208国道过境汽运物流服务中心。提高城市规划、建设、管理水平，深化特色小城镇建设。落实农民工和农民进城购买新建商品住房奖励补贴政策，消化商品住房库存，推进保障性住房建设。完成采煤沉陷区治理搬迁33个村3900户10666人、农村地质灾害治理198户、农村危房改造7690户。抓好美丽宜居示范村“三级联创”和重点古村落保护工作。

　　健全城镇化推进机制。加快户籍制度改革，推行居住证制度。完善城镇基本公共服务，努力实现常住人口全覆盖。落实财政转移支付同农业转移人口市民化、城镇建设用地增加规模同吸纳农业转移人口落户数量挂钩政策。依法维护进城落户农民土地承包权、宅基地使用权、集体收益分配权。

　　加快产业集聚区建设。加快“三个门户”“三个集散地”六大经济板块”建设。进一步做好忻州经济开发区扩区等工作，努力把原平经济技术开发区打造成全市产业集聚发展的排头兵。深入推进全市15个产业聚集区建设，力争落地招商项目45个，项目投资突破100亿元。提高工业园区质量，实现园区承载、循环发展。

　　发展特色现代农业。按照打造知名品牌、壮大龙头企业、培育营销主体、建设产业基地、创新经营模式“五环联动”的思路，全力推动农业内部生产、加工、销售的三次产业分工，着力建设一批带动能力强、辐射范围广、发展后劲大的农产品生产加工大集团、大企业，培育专业种养大户，发展专业家庭农场，壮大农民专业合作社，提升农业产业化、组织化水平。加快农村电子商务发展，提升农产品的市场开拓能力。实施“四化五进”工程，擦亮“中国杂粮之都”名片。落实各项强农惠农富农政策，推进农田水利、土地整治、中低产田改造和高标准农田建设。积极引导农民调整种养结构，多措并举消化粮食库存，按照“市场定价、价补分离”原则，保障农民合理收益。深化农村集体产权制度改革，推进农村土地承包经营权确权登记颁证和国有林场改革，扩大农村土地承包经营权抵押贷款试点范围，激发农村发展活力。

　　推动要素集聚。推进全市优势资源、资产、资金集聚，努力走出去、积极引进来。发挥忻州经济开发区、五台山风景区的示范引领作用，体现忻州发展的高度；把兴县强市作为基本路径，强化目标导向，加快县域内部集聚，推动忻府、原平、代县、繁峙、宁武、河曲、保德等7个县（市、区）加快发展，撑起忻州发展的基本面；推动定襄、五台、静乐、偏关、神池、五寨、岢岚等7个县走好县域经济发展路子，彰显忻州特色。围绕集聚区建设和产业链条各个环节，加快形成人流、物流、资金流、信息流，实现物流、仓储、金融、通讯、电子商务等生产生活服务业配套，以集聚区发展带动经济发展。

　　（五）优化发展环境，打造宜居宜业忻州。

　　持续完善基础设施。做好大同—原平客专建设服务、忻州—五台山—保定客专项目工作；推进晋蒙黄河大桥建设，开通五盂、原神高速，加快神岢高速项目，开展繁五高速项目前期工作；建设五台山机场快速通道，推进6个集中连片特困县的县乡公路改造；完成牧马河忻府区豆罗滚水坝至城区新东门大桥段、峨河繁峙段河道治理续建任务，开工建设牧马河定襄段、朱家川河河曲段河道治理工程，推进大水网和小水网忻州项目区建设。实施配电网标准化建设和新一轮农村电网改造工程，保障3条特高压线过境工程顺利实施，规划实施晋能保德等6条外送线路，全面推进110千伏宁武龙源等5项清洁能源送出工程、110千伏忻府部落等8项骨干电网项目建设。推进大西高铁站前广场、市人民医院、忻州高级中学三个板块开发，启动云中河二期、九龙岗公园、体育广场等项目，实施忻州古城改造和历史文化街区建设，唤醒历史记忆、建设现代新城、体现未来希望。

　　大力改善生态环境。推进多污染物综合防治和环境治理，实行联防联控和流域共治。坚定不移治理大气雾霾。加快锻造业、球团烧结、煤台等重点行业转型升级。强化水污染防治，推进汾河、滹沱河及桑干河上游重点流域生态修复治理，打造汾河源头“三晋水塔”；实施特色小流域治理工程；全面控制重点行业水污染物排放。加强土壤污染防治，严防土壤污染引发食品安全问题。实施林业重点工程，完成营造林70.57万亩，全面提升森林公园、城郊公园、湿地公园建设管理水平。

　　全面提升中介服务。大力发展与项目建设相配套、与产业活动相协调、与城乡居民需求相适应的公证、律师、会计及咨询、招标、拍卖等中介服务机构。全面清理规范行政审批中介服务，督促指导各类中介机构加强自身建设，提升服务水平。

　　切实改善社会环境。开展企业周边地区和突出治安问题专项整治，为项目建设和企业生产经营保驾护航。严厉查处破坏经济发展环境和损害投资者合法权益的不法行为，保护市场主体的合法权益。加大金融诈骗、偷税骗税等案件的查办力度，维护正常的市场秩序。

　　有效改善政务环境。推动“放管服”改革向纵深发展，不断转变职能、提高效能。如期公布县级政府权力清单和责任清单，实行目录清单管理；推进注册登记便利化，深化“先照后证”改革；实施“互联网+政务服务”，加快“一网络两平台”建设，建成运行市电子政务平台。提升“忻州随手拍”“忻州发布”的运行水平，实现网络问政常态化。

　　着力建设诚信忻州。基础设施是环境，环保生态是环境，中介服务是环境，社会秩序是环境，政务服务是环境，诚信是更重要的环境。建立和完善诚信体系，加快建立统一的社会信用平台，启用市场主体信用信息系统，深化诚信县、乡、村、单位、企业创建活动。发挥干部带头示范作用，身体力行，有诺必践，以诚信的作风取信于民。大力倡导契约精神，不断强化信用约束，着力解决不诚不信的突出问题，让失信者一处违法、处处受限，让守信者受到尊重、一路畅通，切实增强全社会的诚信意识和信用观念，努力在全市上下形成人人讲诚信、个个守信用的浓厚氛围，使诚实守信成为忻州的金字招牌。

　　（六）加强文化建设，凝聚推动发展的强大正能量。

　　培育社会主义核心价值观。加强新闻舆论工作，大力弘扬改革创新的时代精神，用发展理念凝聚共识、汇聚正能量。充分发挥社会主义先进文化、优秀传统文化、红色文化资政育人作用，深入开展社会公德、职业道德、家庭美德和个人品德教育。深化群众性精神文明创建活动，倡导全民阅读，普及科学知识，提升全民素质和社会文明程度。

　　构建公共文化服务体系。加快市级“五馆一院”建设，继续推动县级“三馆一院”建设，建设“数字化档案”。加强文化艺术人才队伍建设和文化市场管理，深化公益性文化事业单位内部改革。引导公共文化资源向城乡基层倾斜，推进数字广播电视户户通和乡村文化记忆工程。加强文化遗产保护利用。

　　繁荣文化事业、发展文化产业。不断促进文化与金融、旅游、科技的融合发展，推进文化创意产业发展。坚持以人民为中心的创造导向，鼓励特色精品创作。促进传统媒体与新兴媒体融合发展。加强网上思想文化阵地建设，净化网络环境。实施好“送戏下乡”等文化惠民服务。倡导全民健身，提高竞技体育水平。

　　（七）切实保障改善民生，努力造福群众。

　　着力稳定和扩大就业。实施更加积极的就业政策，鼓励以创业带动就业。落实大学生就业促进计划和创业引领计划，发挥大学生创业园的孵化作用，继续安排3000万元全民创业专项扶持资金和3000万元中小企业发展专项资金，推动大众创业、万众创新。加快发展劳动密集型产业，多渠道开发就业岗位。统筹做好农村转移劳动力、城镇失业人员、退役军人等群体的就业工作，引导和支持企业在化解过剩产能、克服运行困难过程中稳定就业岗位。继续实行政府购买基层公共服务岗位、招聘农村特岗教师、“三支一扶”等措施，吸纳高校毕业生就业。切实做好退役军人安置和就业创业服务工作。实施职业培训全覆盖计划。

　　努力增加群众收入。健全企业工资增长机制，兑现机关事业单位工作人员调资政策，落实县以下机关公务员职务职级并行制度和乡镇工作补贴，健全农民工工资支付保障机制，增加农民转移性收入、工资性收入、财产性收入。

　　完善社会保障体系。稳步推进机关事业单位、企业基本养老保险制度并轨运行，提高退休人员基本养老金待遇水平。建立统一的城乡居民基本医疗保险制度，财政补助由每人每年380元提高到420元。合并实施基本医疗保险和生育保险。完善重特大疾病医疗救助政策。落实经济困难的高龄与失能老年人补贴以及百岁以上老年人补贴新标准，全面实施困难残疾人生活补贴和重度残疾人护理补贴制度，加快健全城乡社会救助体系，确保困难群众遇急有助、遇困有帮。

　　大力发展社会事业。扩大学前教育资源，完成河曲、代县、五寨、宁武4县的“全面改薄”年度任务，如期完成忻州高级中学、长征小学、实验幼儿园项目建设，办好特殊教育，发展现代职业教育，落实提高乡村教师待遇政策。启动新建忻州技师学院，筹备忻州旅游学院、第十四中学、雁门小学以及忻州十二中、实验二校改造项目前期工作，为明年全面开工奠定基础。加快县级公立医院改革，完善基本药物制度和公立医院药品采购机制，推进分级诊疗制度建设，强化重大疾病防控。基本公共卫生服务经费财政补助从人均40元提高到45元。加快市儿童医院、药检中心、农产品检测中心、残疾人康复中心和托养中心建设。实施一对夫妇可生育两个孩子政策。做好妇女、儿童、外事、侨务、气象、地震、老龄、人民防空等工作。

　　重视公众心理疏导。着力增强群众工作本领，加强对社会热点难点问题的引导，解决好涉及群众切身利益的问题，稳定群众心理预期。落实重大决策社会稳定风险评估机制，有效预防和化解矛盾。深化普法教育，强化群众法治意识。

　　加强和创新社会治理。创新城乡基层群众自治和社区治理，推进城镇社区“网格化”管理。关爱农村留守儿童、妇女和老人，加强未成年人保护。改进信访、人民调解工作。创新立体化社会治安防控体系。健全社会信用体系，加强食品药品安全监督执法，推进可追溯体系和检验检测体系建设。推开“两随机一公开”监管。

　　（八）实施脱贫攻坚工程，加快全面建成小康社会进程。

　　夯实增收产业基础。开展35个乡1万户3万人的产业扶贫试点。推进企业产业扶贫，加大岢岚周通、五台中扶惠帮等农业综合项目开发带动力度，实施光伏扶贫集体收益倍增试点，开辟贫困村、贫困人口增收渠道。推动 “百企千村”产业扶贫项目重新启动。实施生态产业，开展小流域治理，让工商资本进入治山治水的小流域治理领域，推动生态治理和群众脱贫致富融合发展。实施金融扶贫富民工程，年内发放各类扶贫贷款3亿元，有效支持有产业开发需求的贫困户。坚持群众主体，发挥基层作用，依法依规加强资金分配管理，努力走出扶贫资金整合使用新路子。

　　实施易地扶贫搬迁。认真制定搬迁规划和方案，科学确定集中安置项目主体，依法依规加强资金管理，充分发挥基层组织和群众的作用，易地扶贫搬迁9170户24114人。统筹贫困群众搬迁安置和生产生活，优先向移民新村安排扶贫开发项目。全年完成8万贫困人口脱贫任务。

　　开展技能培训。坚持扶贫与扶志、扶智相结合，制定培训计划，落实培训基地，强化现场培训，发挥岢岚县羊产业、定襄县种植业和忻府区、保德县、五台县乡村旅游业的示范作用，完成4000人的新型职业农民培育和6600人的千村万人就业培训任务，使贫困人口掌握一技之长，切实提高自我发展的能生动力和能力。

　　改善生产生活条件。用足用好政策，鼓励探索创新，完善基础设施和公共服务设施，保障贫困群众基本生产生活需要。加大建档立卡贫困家庭教育扶贫力度。推动低保线和贫困线“两线合一”。加大贫困人口医疗救助、临时救助和社会救助力度，确保政策兜底户首批实现脱贫。

　　（九）毫不放松抓好安全生产，保障人民生命财产安全。

　　深化安全发展理念。牢固树立红线意识、忧患意识和责任意识，以铁的担当尽责、铁的手腕治患、铁的办法治本，为塑造忻州美好形象、实现忻州振兴崛起营造安全稳定环境。加强安全生产法治建设，认真落实《安全生产法》。深入落实“4438”机制，坚持完善系列行之有效的制度措施，不断强化安全生产的法律和制度保障。

　　落实安全生产责任。按照“党政同责、一岗双责、失职追责”的要求，切实做到管行业必须管安全、管业务必须管安全、管生产经营必须管安全，以政府领导责任、部门监管责任的落实推动企业主体责任的落实。

　　深化隐患排查治理。完善安全生产风险预控和隐患排查治理双重预防机制。引深安全生产大检查行动，采用“四不两直”等方式，全面加强煤矿、道路交通、非煤矿山和尾矿库、危化品、人员密集场所以及建筑施工、油气管道、城市燃气、特种设备等行业和领域安全隐患排查整治，坚决打击非法违法生产经营建设行为。

　　强化问责追究。严格落实目标责任考核“一票否决制”，继续实施企业重大隐患和事故挂牌督办及“黑名单”制度。严肃查处事故，严格责任追究，坚决杜绝重特大事故、有效遏制较大事故、着力减少一般性事故，促进安全生产形势由稳定好转向根本好转的目标迈进。

　　（十）加强政府自身建设，提升施政能力和服务水平。

　　坚持执政为民。扎实开展“两学一做”学习教育，牢固树立“四个意识”，加强作风和能力建设，努力打造法治、为民、服务、效能、诚信、廉洁的“六型”政府。

　　坚持从严治政。严格落实全面从严治党要求，持续加强政府的廉政建设。严格执行中央八项规定精神和国务院“约法三章”，持续狠刹“四风”，厉行勤俭节约，严控“三公”经费，切实做到为民、务实、清廉，做勇于决断担当的表率、廉洁从政的表率、导扬风化的表率。

　　坚持依法行政。进一步转变政府职能，持续推进行政审批制度改革。严明纪律，严守规矩，确保政府一切行为在法律和政策范围内活动。坚持自觉运用法治思维和法治手段解决问题、推动工作。推行政府法律顾问制度。加强行政监察，完善审计制度，做好行政复议。启动“七五”普法，强化法律援助。

　　坚持公开公正。自觉接受人大及其常委会的监督，主动接受政协的民主监督，认真听取民主党派、工商联、无党派人士和人民团体的意见，重视司法、舆论、公众监督，让权力在阳光下运行。深入推进政务公开，充分发挥传统媒体、新兴媒体作用，强化问题跟踪督办，积极回应社会关切。

　　坚持求实求是。把问题作为时代的声音、前进的起点，深入调查研究，吃透真实情况，努力提升政府系统工作人员发现问题、解剖问题、扭住根本问题的能力；深入研究政策，把握发展趋势，努力提升政府系统工作人员掌握规则、适应规则、应用规则解决问题的能力。

　　坚持真抓实干。紧紧围绕经济社会发展目标，引导干部群众适应新语境，进入新语境，提升思想境界，保持创新追求，做到担难、担险、担诺、担远、担责。加强常态化督查，做到有部署就有督查、有督查就有结果，推动政府工作人员知责、履责、尽责。

　　各位代表！今天的奋斗必将成就明天的光荣。让我们在市委的坚强领导下，认真实施“1661”发展战略，孜孜以求，真抓实干，努力完成今年经济社会发展目标任务，实现“十三五”良好开局，为塑造忻州美好形象，实现忻州振兴崛起，全面建成小康社会而努力奋斗！
